# Supplementary material for: Ayu: a machine intelligence tool for identification of extracellular proteins in the marine secretome
Source: Nat Commun. 2025 Mar 21;16:2793. doi: 10.1038/s41467-025-57974-5 (PMC11928666; doi:10.1038/s41467-025-57974-5)
Supplement: Supplementary file 1 — Supplementary Information [file 41467_2025_57974_MOESM1_ESM.pdf]

Supplementary Information for "Ayu: A machine intelligence tool for identification of extracellular proteins in the marine secretome"

Asier Zaragoza-Solas, Federico Baltar

Includes:

- Supplementary figures S1-S14
- Supplementary text S1-S2

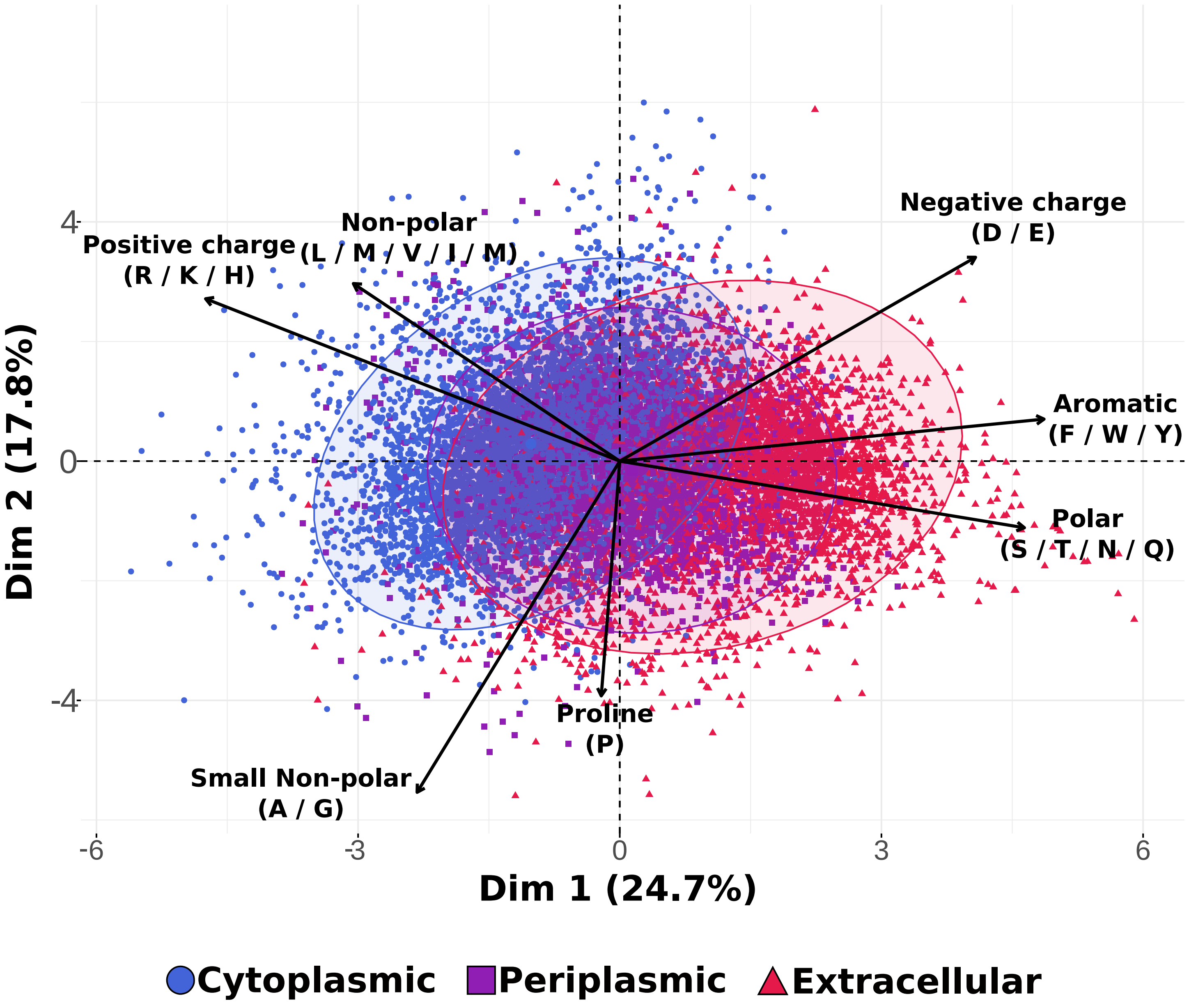

Figure S1: **Differences in amino acids between proteins based on subcellular location**. Weighted log-ratio biplot of the marine protein dataset, based on ratios of grouped amino acids. Each protein is shaped and coloured according to their subcellular location.

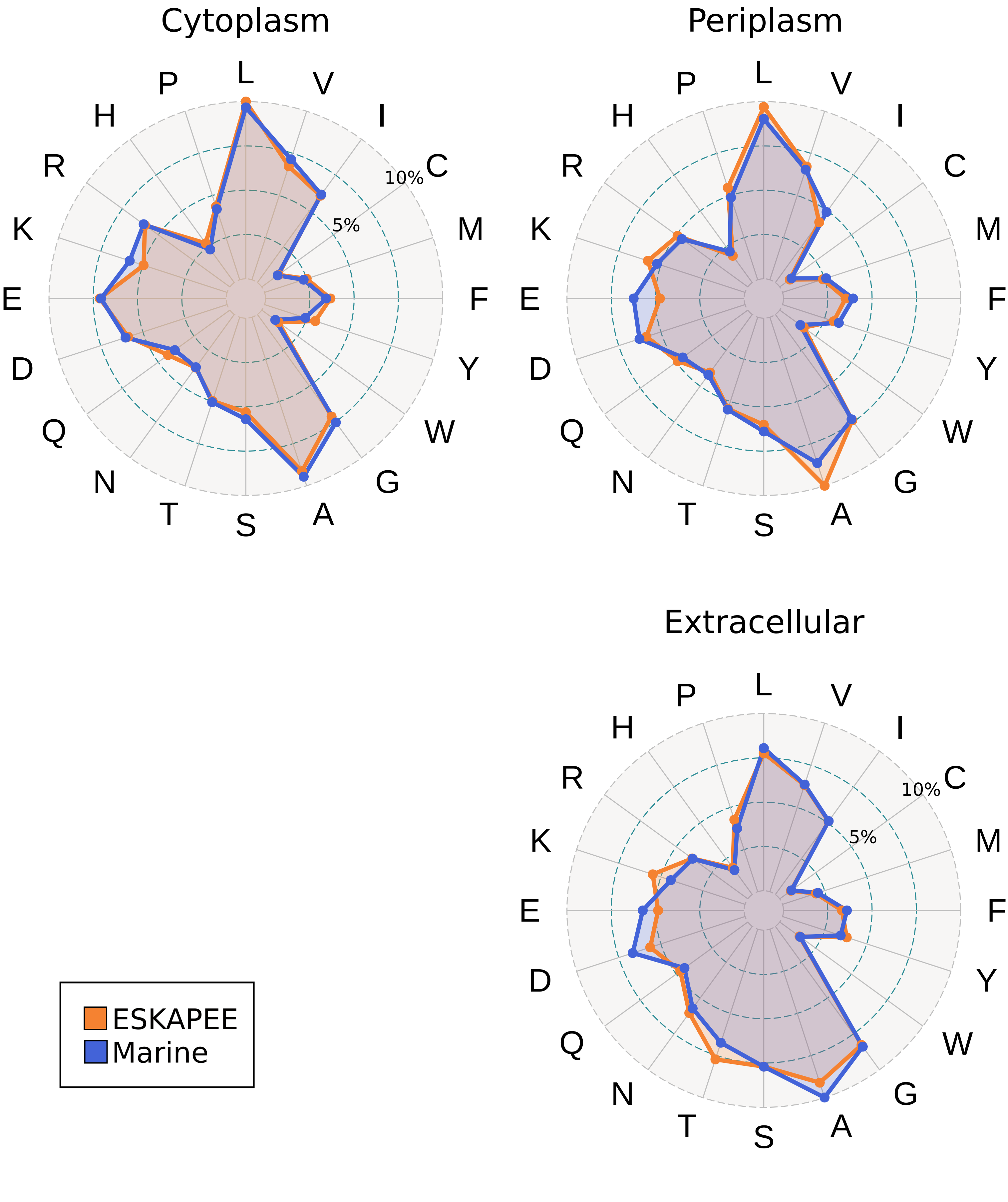

Figure S2. **Differences in amino acid composition based on habitat.** Radar plots of amino acid composition for the marine (blue) and ESKAPEE (orange) protein datasets, separated by cellular sublocation.

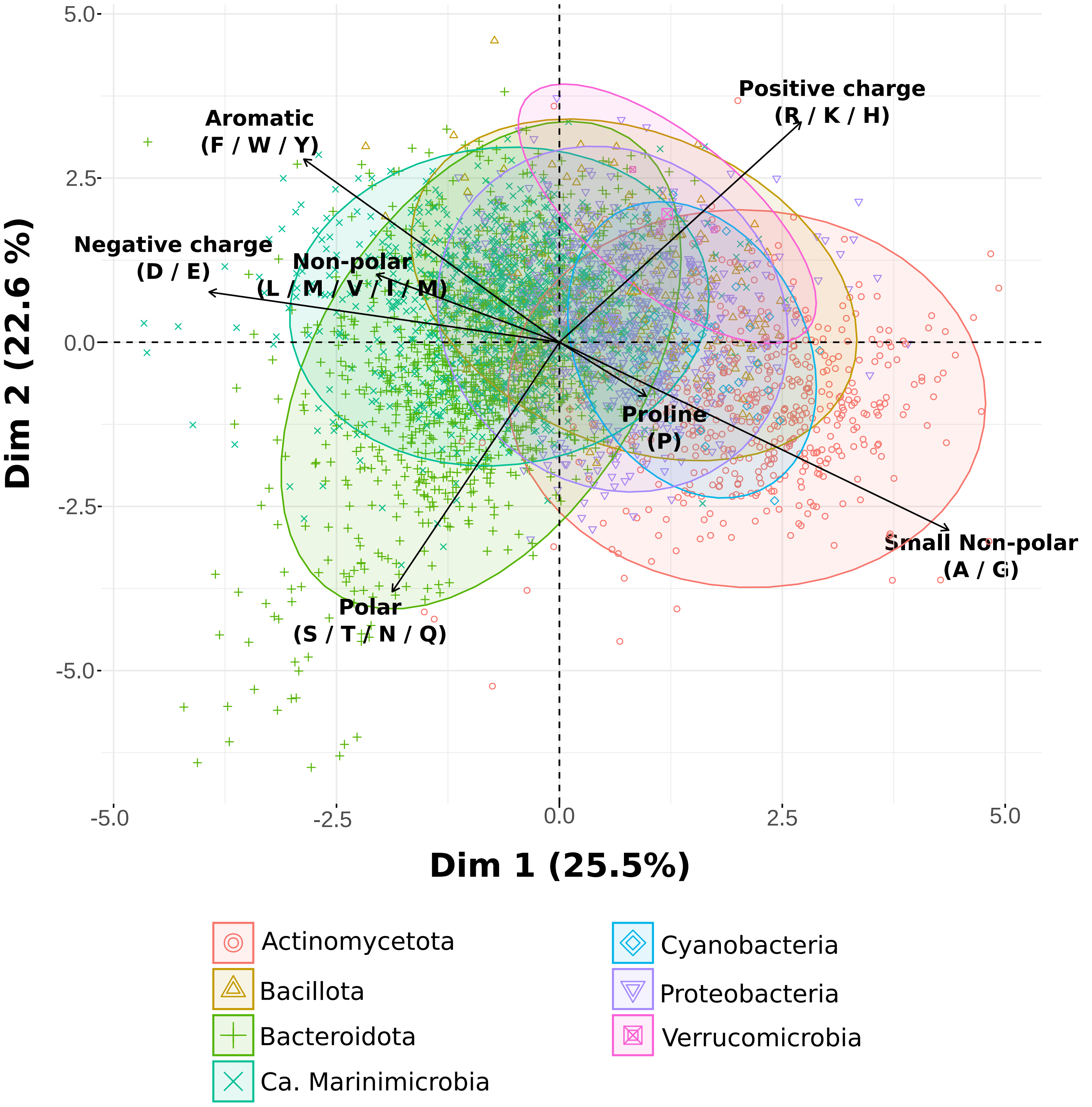

Figure S3. Differences in amino acid composition based on Taxonomy. Weighted log-ratio biplot of the extracellular portion of the marine protein dataset, based on ratios of grouped amino acids. Each protein is shaped and coloured according to their taxonomic class.

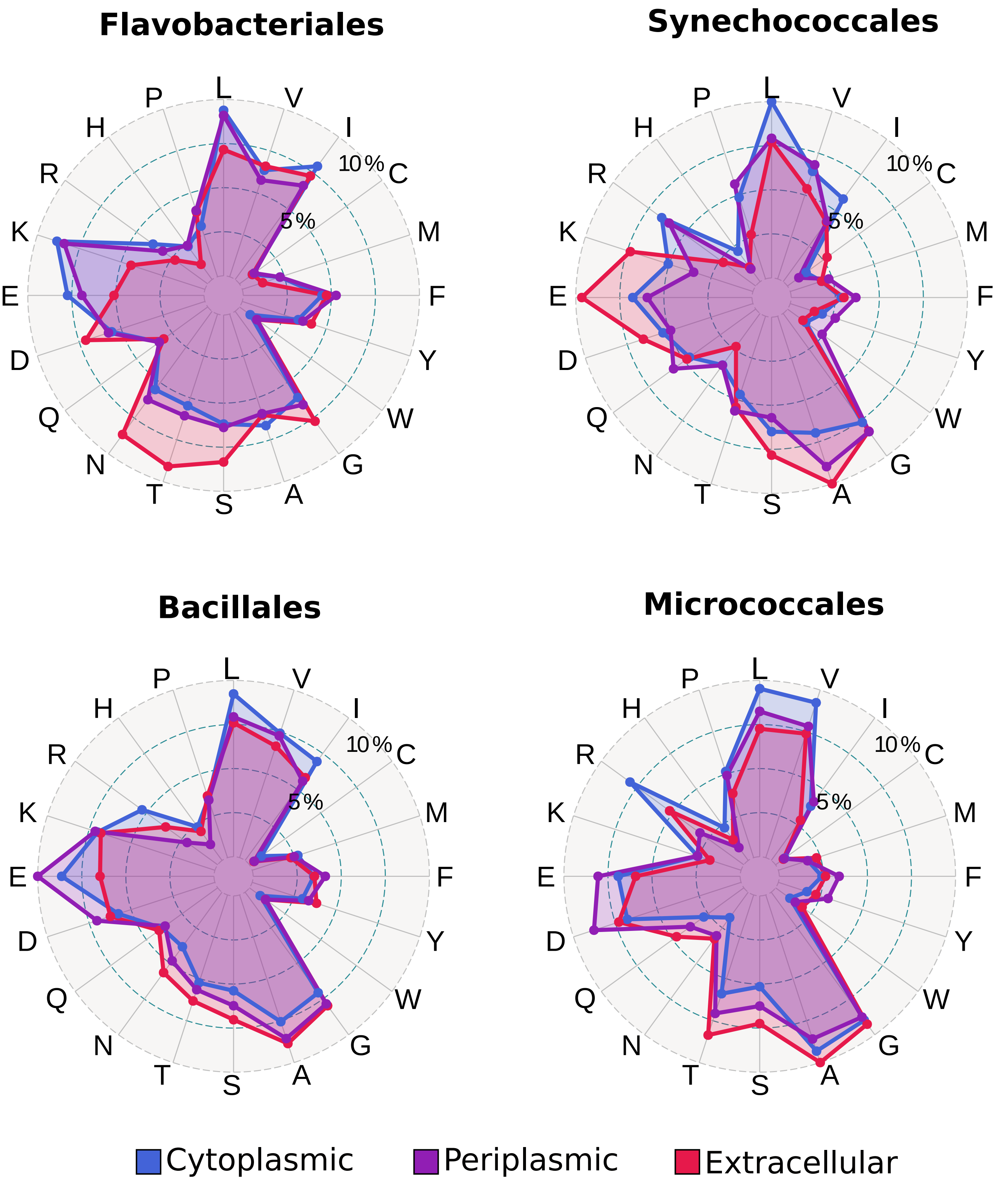

Figure S4. **Amino acid composition of proteins by taxonomy.** Radar plots of amino acid composition for the marine protein datasets, separated by subcellular location and taxonomic class.

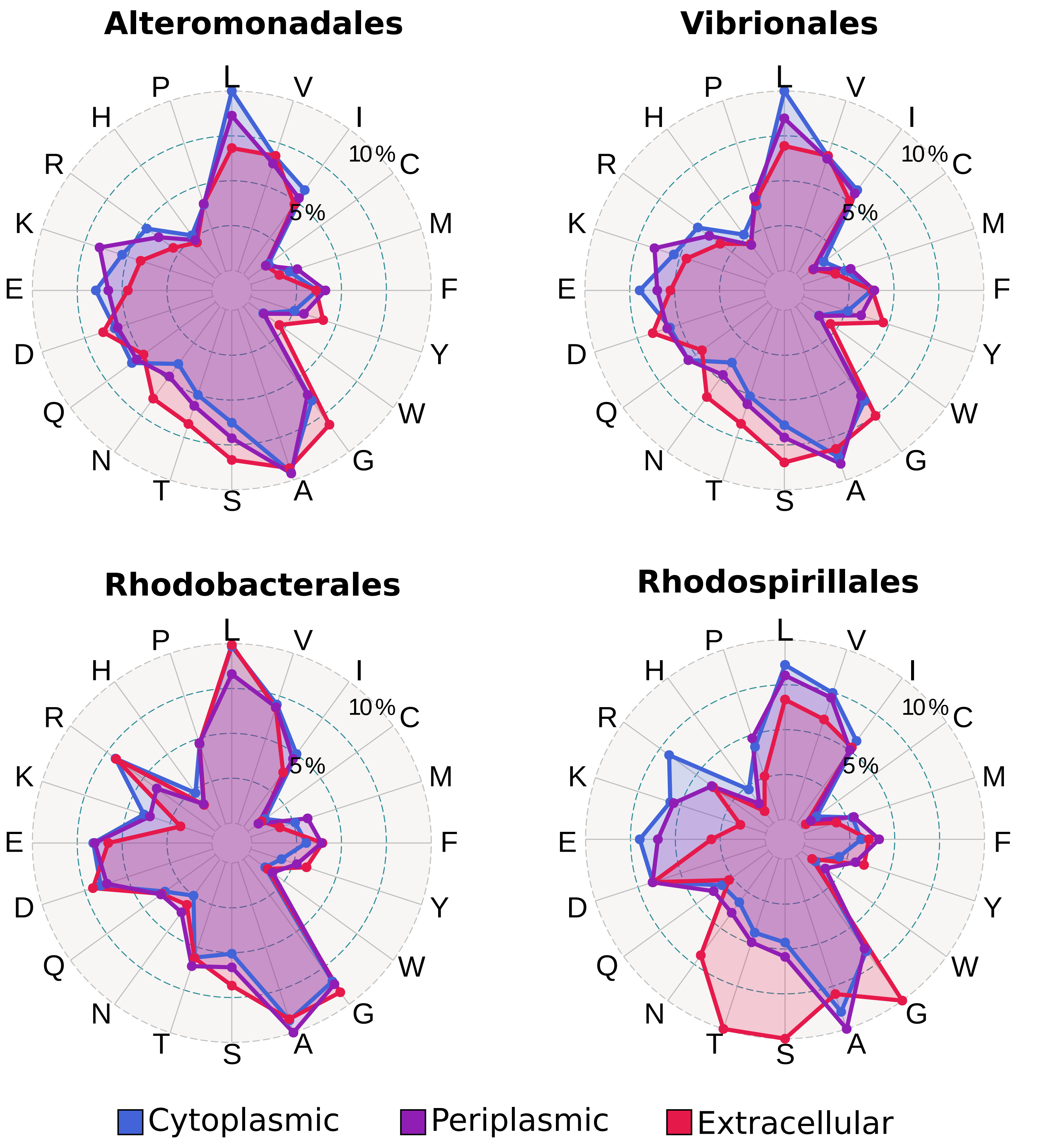

Figure S5. **Amino acid composition of proteins by taxonomy.** Radar plots of amino acid composition for the marine protein datasets, separated by subcellular location and taxonomic class.

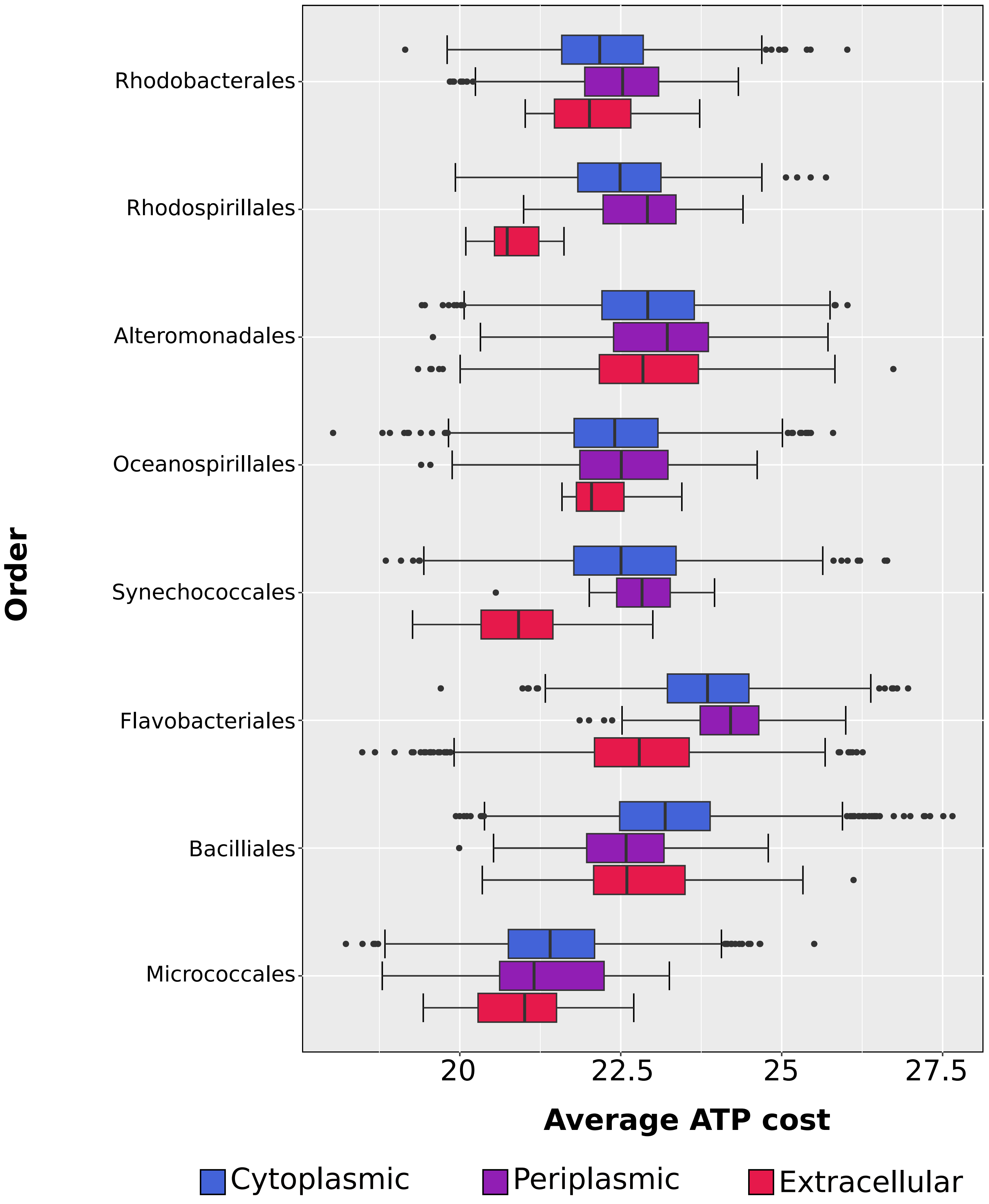

Figure S6. **ATP Cost of marine proteins.** Average ATP cost of proteins from the marine dataset, separated by taxonomical order and subcellular location. In the box plots, the black bar indicates the median, the range of each box extends from the first to the third quartile, and whiskers extend to 1.5-fold interquartile range.

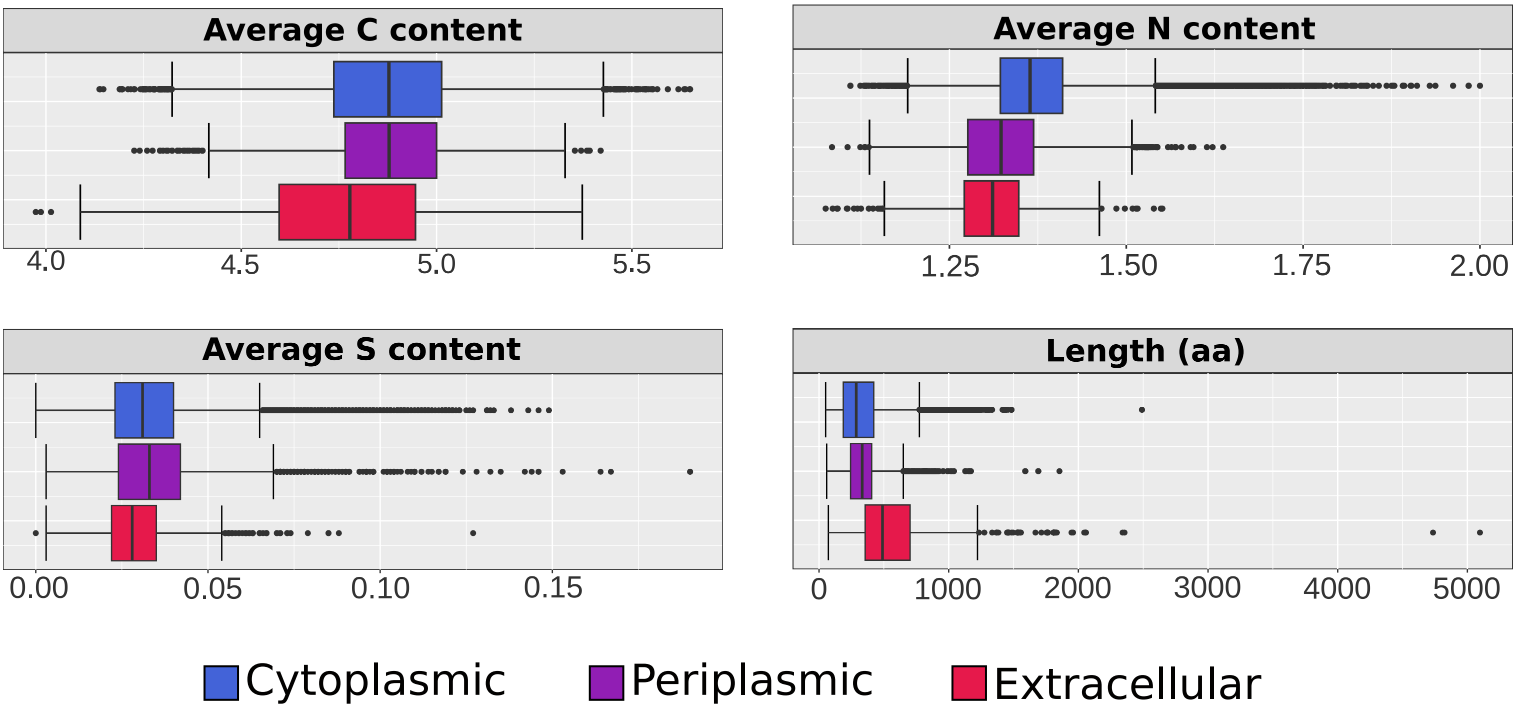

Figure S7. **Physicochemical values of marine proteins.** Distributions of physicochemical values (average carbon content, average nitrogen content, average sulphur content and length) derived from proteins from the marine dataset, separated by subcellular location. In the box plots, the black bar indicates the median, the range of each box extends from the first to the third quartile, and whiskers extend to 1.5-fold interquartile range.

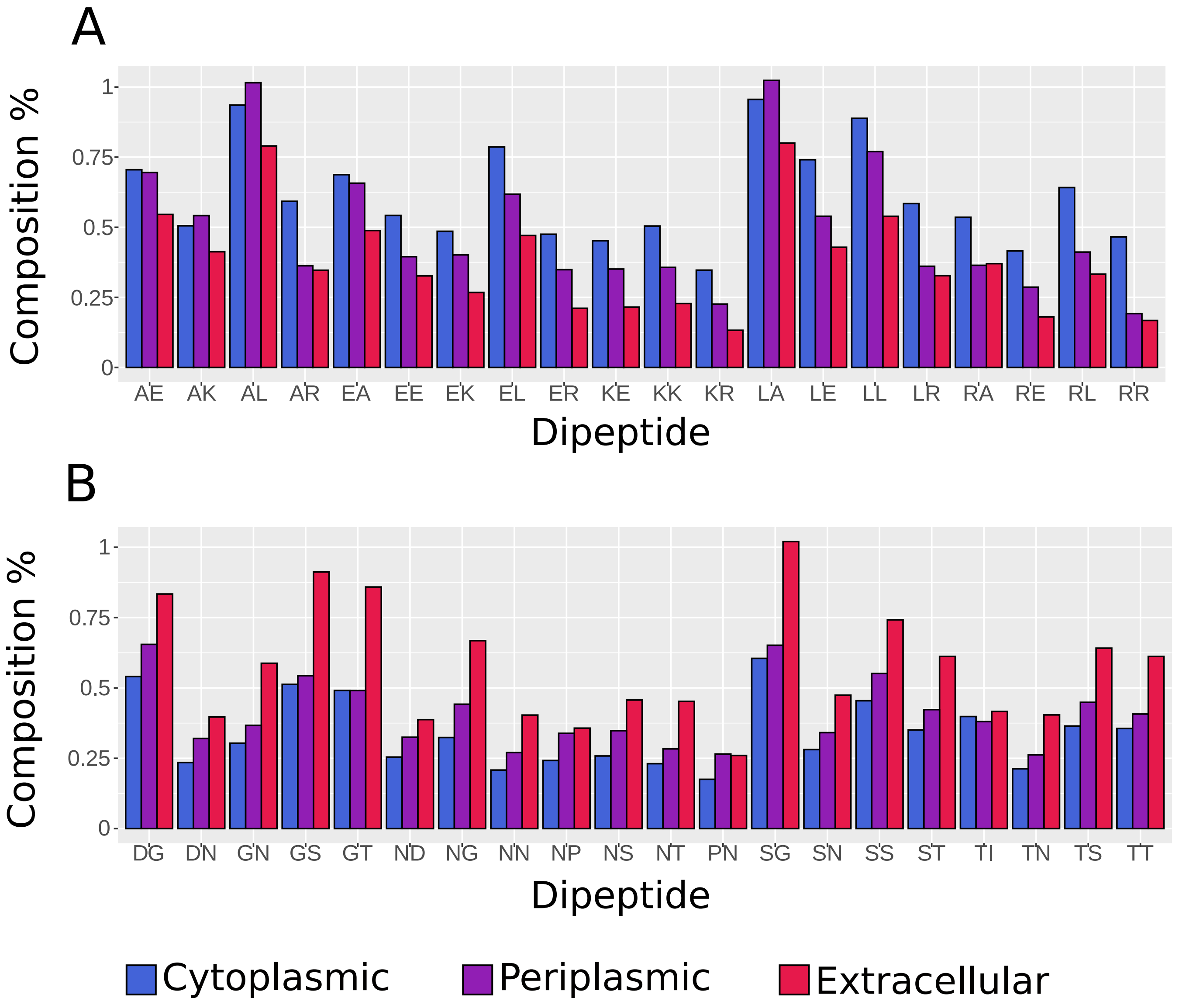

Figure S8. Most abundant dipeptide combinations by subcellular location. Dipeptide ratios most abundant in cytoplasmic (A) and extracellular (B) proteins, separated by subcellular location.

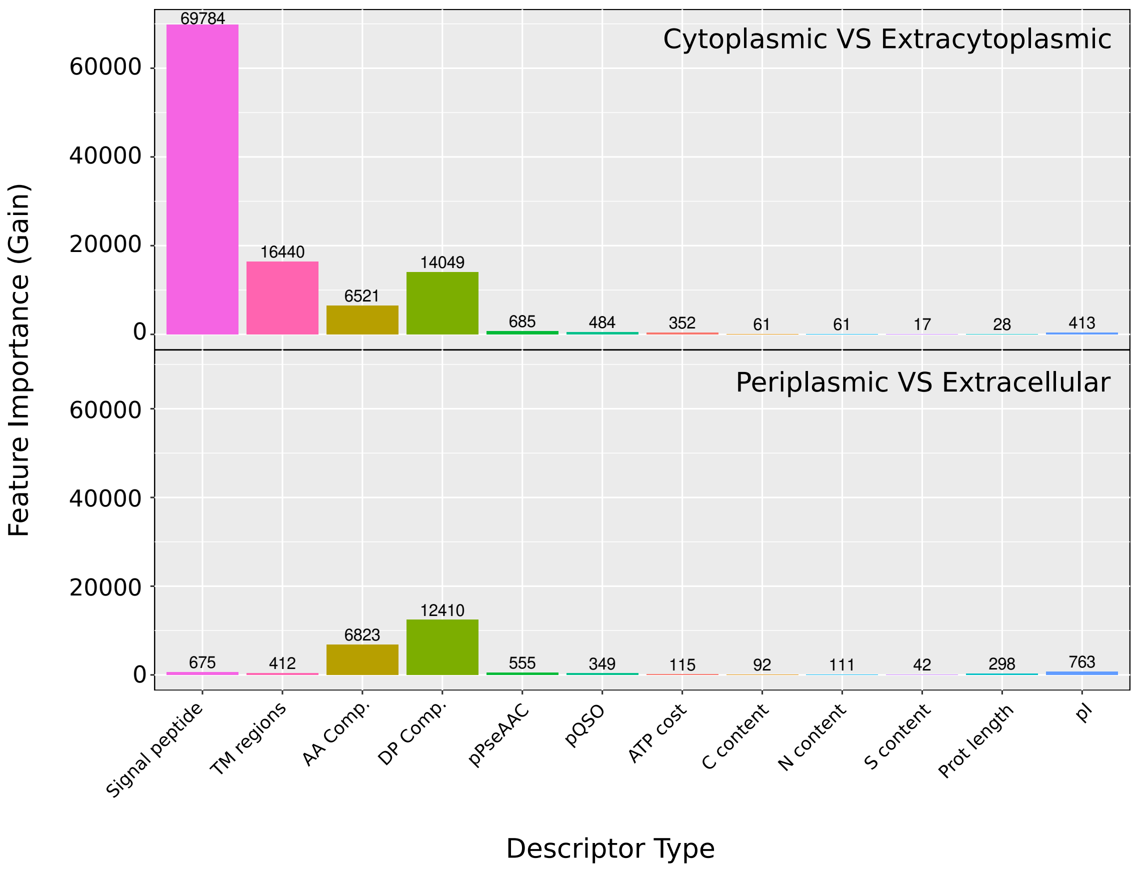

Figure S9. Gain values for the Ayu ordinal classifier. Feature Importance (Gain) for both elements of the Ayu ordinal classifier.

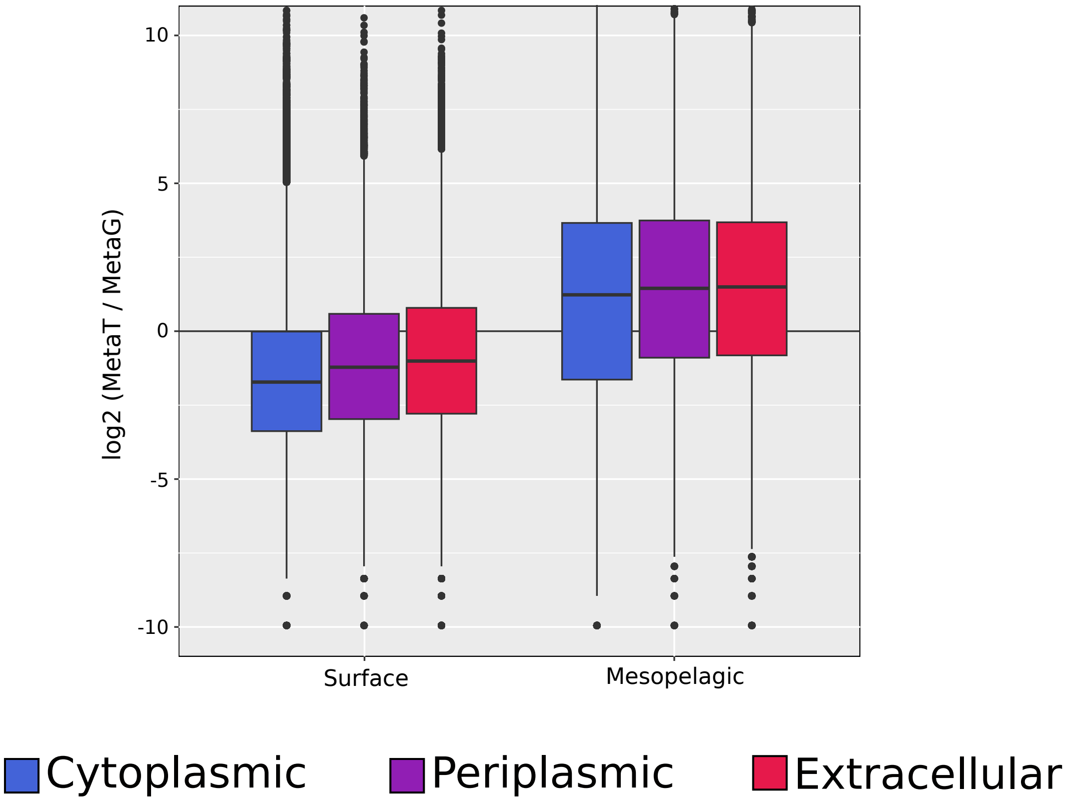

Figure S10. **Ratios of metagenome to metatranscriptome in TARA Oceans.** log2 ratios of RPKG in metagenome vs RPKG in transcriptome for Tara oceans bacterial proteins, separated by subcellular location. (n=8,674). In the box plots, the black bar indicates the median, the range of each box extends from the first to the third quartile, and whiskers extend to 1.5-fold interquartile range.

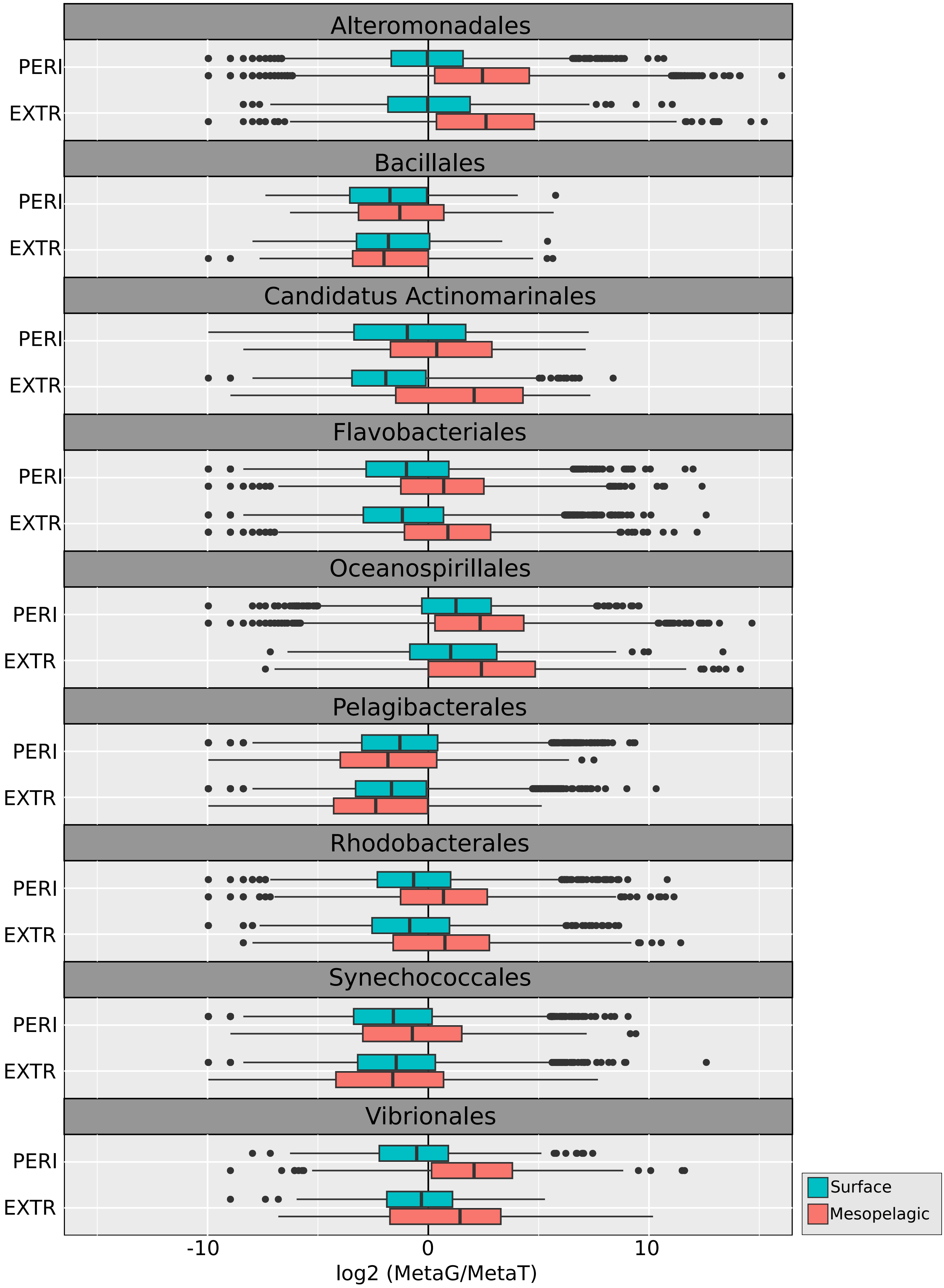

Figure S11. Log ratios of metagenome / metatranscriptome in Tara Oceans. log2 ratios of RPKG in metagenome vs RPKG in transcriptome for Tara oceans bacterial proteins (n = 8,674), separated by subcellular location, depth and taxonomic clade. In the box plots, the black bar indicates the median, the range of each box extends from the first to the third quartile, and whiskers extend to 1.5-fold interquartile range.

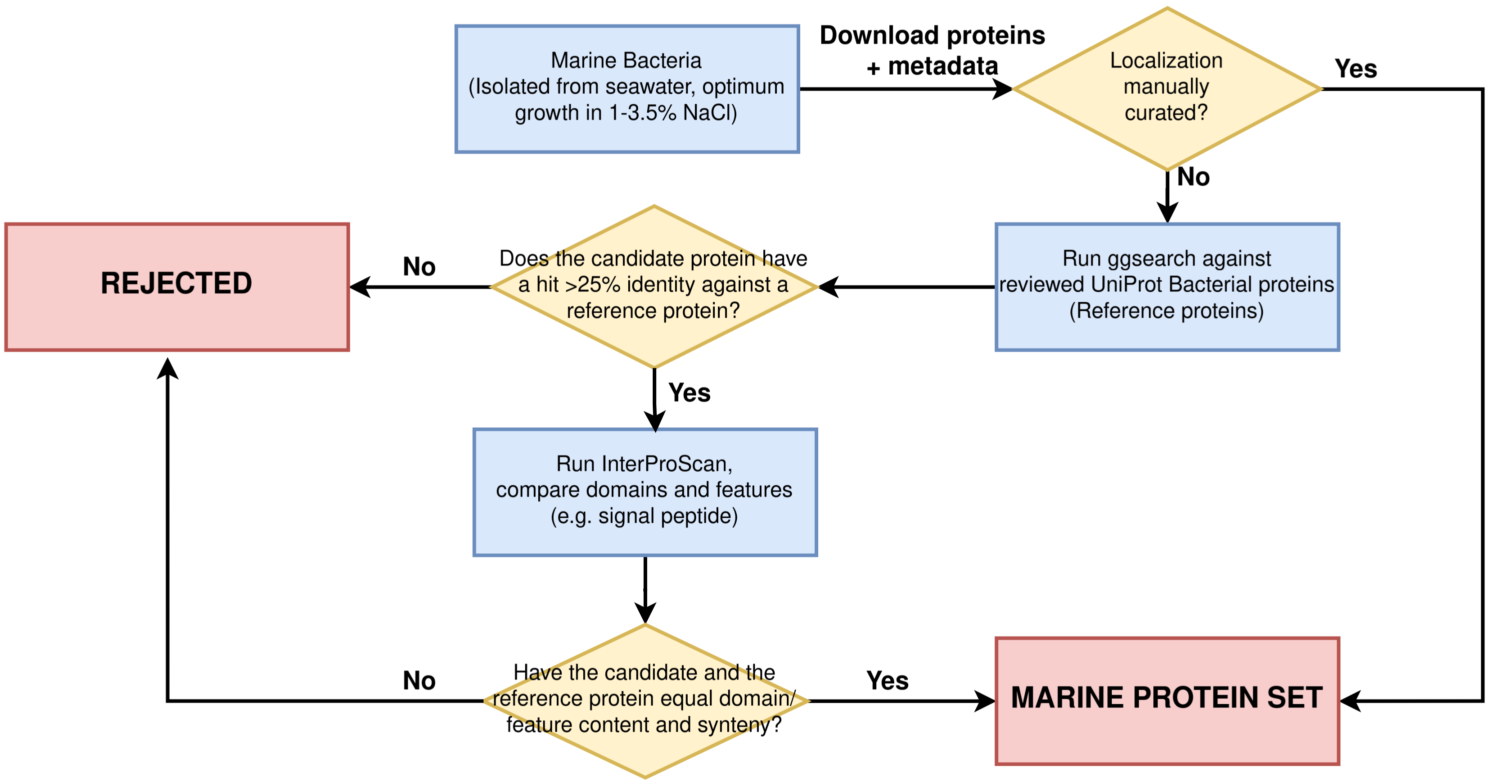

Figure S12. Data collection workflow. Data collection workflow for the training and validation datasets.

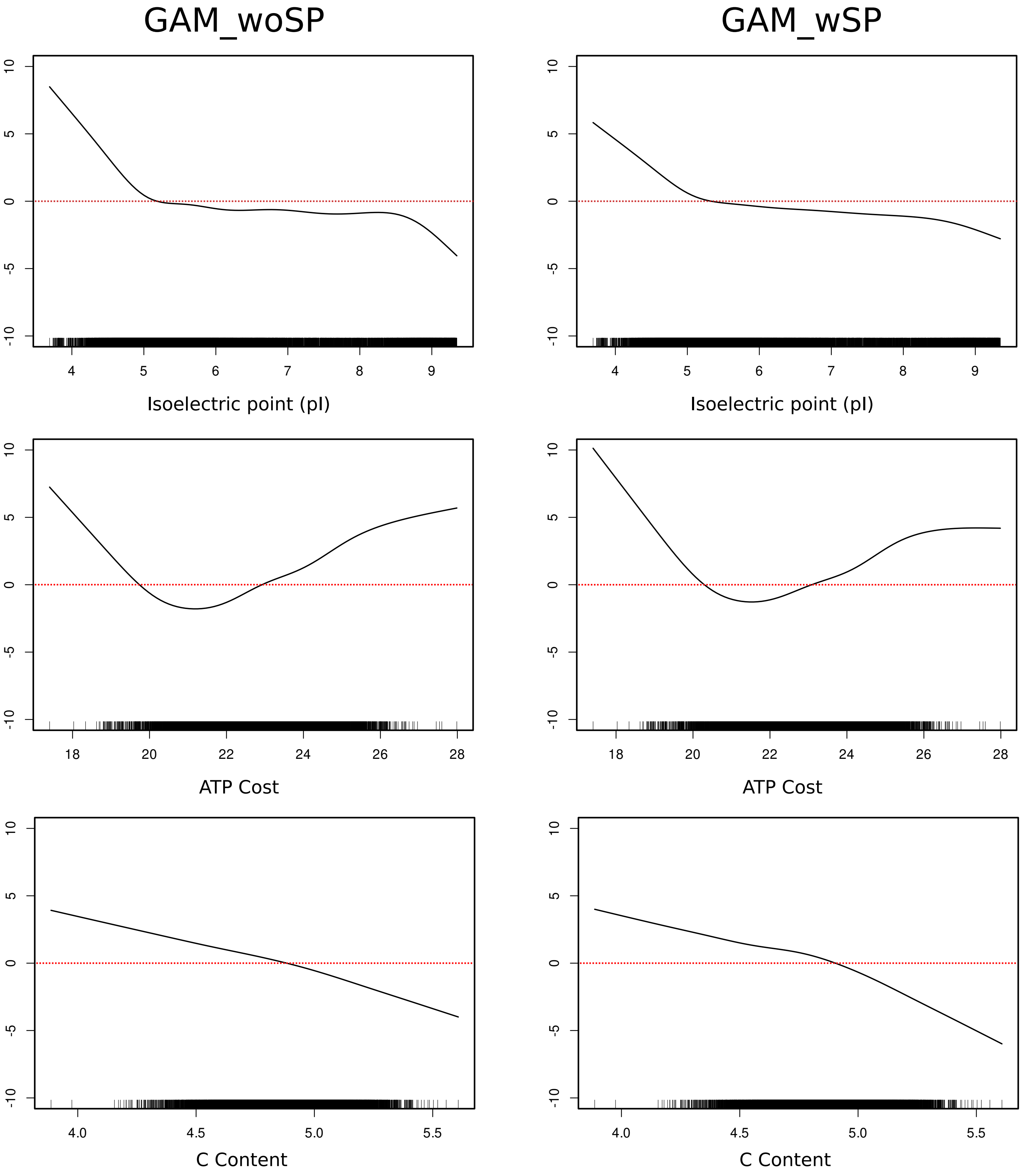

Figure S13. Feature shapes of the marine protein dataset. Feature shapes of Generalized Additive Models (GAMs) trained on the marine protein dataset. GAM_wSP is a GAM including signal peptide information plus all other features, GAM_woSP does not include the signal peptide feature. The dotted red line indicates the position in which the feature is not contributin gto the model.

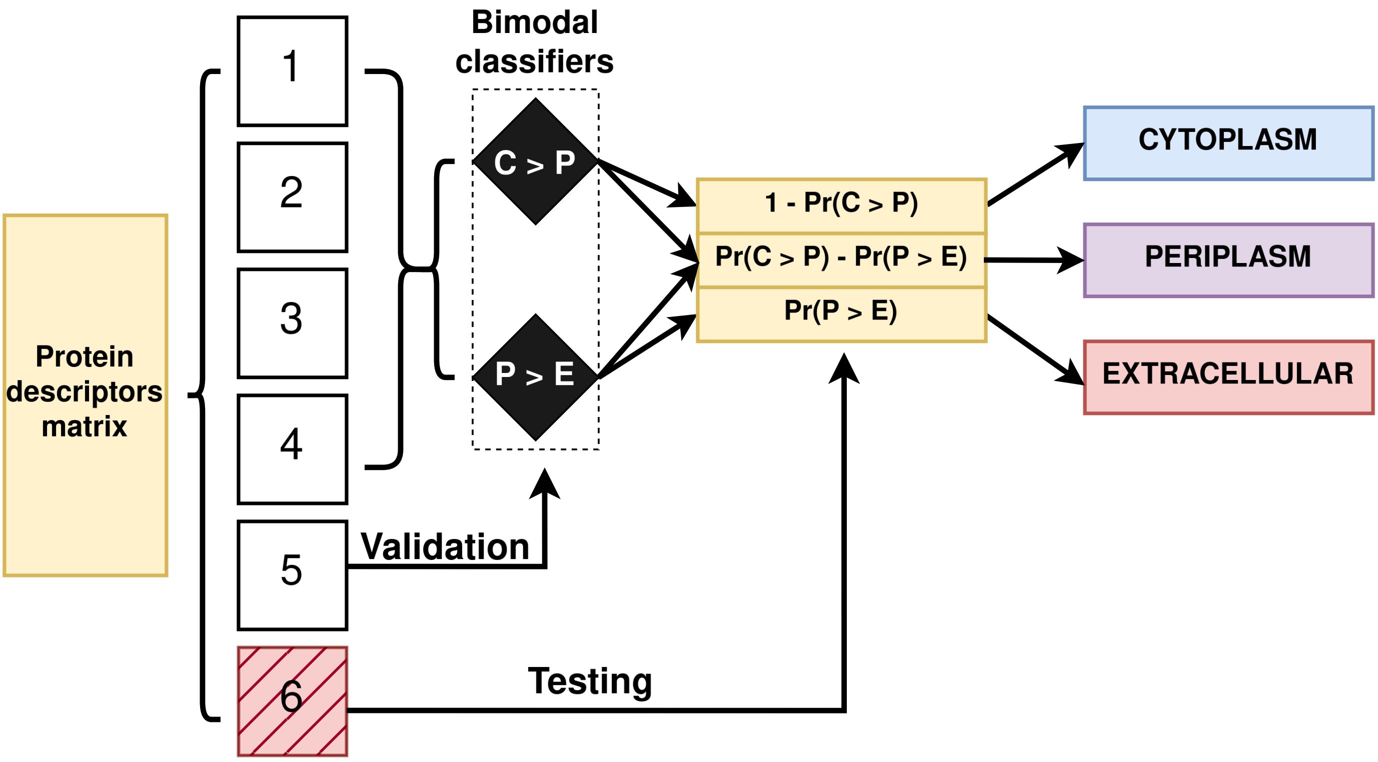

Figure S14. Model for ayu ordinal classifier. The final model is composed of two binary classificators: the first classifies proteins into cytosolic or extracytosolic (C > P), while the second classifies proteins into periplasmic or extracellular (P > E). The probabilities for each of the two classifiers are then combined to obtain the final probabilities for each of the three cellular locations.

Supplementary text for “Ayu: prediction of extracellular proteins in large marine datasets by exploiting adaptations to the marine environment”

Supplementary Text 1: Complete output of Dirichlet regression

Formula: aacomp_data ~ subloc + cellwall_type + dataset_type + subloc:dataset_type

aacomp_data: Amino acid composition of analysed proteins
cellwall_type: Cell wall type of the organism that codes for the protein (Gram +, Gram -)
dataset_type: Type of organism group (Either Marine or ESKAPEE)
subloc: Cellular location (In this case, CYTO: cytoplasmic, PERI:periplasmic, EXTR: Extracellular)
subloc:dataset_type: Interaction, in this case how the combination of cellular location and organism group affect amino acid composition.

```
Standardized Residuals:
             Min       1Q   Median       3Q      Max
Comp_A  -3.5796  -0.8326  -0.0042  0.9769   9.3481
Comp_C  -1.2384  -0.7483  -0.3233  0.2207  10.0948
Comp_D  -2.6568  -0.5282  -0.0845  0.3655   4.9079
Comp_E  -2.9038  -0.6222  -0.1077  0.4782   6.6279
Comp_F  -2.2279  -0.6019  -0.1088  0.4402   7.2041
Comp_G  -3.2157  -0.6173  -0.0574  0.5501   5.8206
Comp_H  -1.8538  -0.6268  -0.1557  0.3875   9.2080
Comp_I  -2.6038  -0.7112  -0.0803  0.6482   5.9962
Comp_K  -2.6685  -0.8718   0.0547  1.2201  10.6122
Comp_L  -3.4478  -0.5606  -0.0521  0.5012   3.9874
Comp_M  -1.7887  -0.6313  -0.2221  0.2731   9.7557
Comp_N  -2.6191  -0.7105  -0.0664  0.6891   6.7710
Comp_P  -2.5262  -0.5911  -0.0681  0.4984  11.1093
Comp_Q  -2.4693  -0.6924  -0.1486  0.5618   9.1771
Comp_R  -2.6354  -0.7720  -0.0695  0.7833  10.3535
Comp_S  -2.9012  -0.6116  -0.1050  0.4573   5.4723
Comp_T  -2.7042  -0.5697  -0.1063  0.3798   5.0076
Comp_V  -2.9194  -0.6133  -0.0850  0.4851   4.9419
Comp_W  -1.3536  -0.7780  -0.3376  0.2574   4.4348
Comp_Y  -2.0325  -0.6487  -0.0962  0.5022   4.9412

MEAN MODELS:
-----------------------------------------------------------------
Coefficients for variable no. 1: Comp_A
                          Estimate Std. Error z value Pr(>|z|)
(Intercept)                0.64417    0.02047  31.472  < 2e-16 ***
sublocEXTR                -0.09463    0.03264  -2.899  0.00374 **
sublocPERI                 0.19797    0.03048   6.496 8.26e-11 ***
cellwall_typeGRAM_P        0.21837    0.01286  16.987  < 2e-16 ***
dataset_type1              0.19310    0.02076   9.300  < 2e-16 ***
sublocEXTR:dataset_type1 -0.01457    0.03704  -0.393  0.69399
sublocPERI:dataset_type1 -0.23435    0.03258  -7.193 6.36e-13 ***
-----------------------------------------------------------------
Coefficients for variable no. 2: Comp_C
```

```
                         Estimate Std. Error z value Pr(>|z|)
(Intercept)              -1.19663    0.03201 -37.379  < 2e-16 ***
sublocEXTR               -0.30907    0.05218  -5.923 3.17e-09 ***
sublocPERI               -0.59289    0.05354 -11.073  < 2e-16 ***
cellwall_typeGRAM_P      -0.07356    0.01991  -3.695  0.00022 ***
dataset_type1             0.09872    0.03247   3.040  0.00236 **
sublocEXTR:dataset_type1 -0.10157    0.05989  -1.696  0.08988 .
sublocPERI:dataset_type1  0.12445    0.05716   2.177  0.02946 *
------------------------------------------------------------------
Coefficients for variable no. 3: Comp_D
                          Estimate Std. Error z value Pr(>|z|)
(Intercept)               0.257803   0.021792  11.830  < 2e-16 ***
sublocEXTR               -0.127537   0.035195  -3.624  0.00029 ***
sublocPERI               -0.081392   0.033537  -2.427  0.01523 *
cellwall_typeGRAM_P       0.202116   0.013557  14.909  < 2e-16 ***
dataset_type1             0.199031   0.022138   8.990  < 2e-16 ***
sublocEXTR:dataset_type1  0.052221   0.039809   1.312  0.18959
sublocPERI:dataset_type1 -0.008196   0.035779  -0.229  0.81881
------------------------------------------------------------------
Coefficients for variable no. 4: Comp_E
                         Estimate Std. Error z value Pr(>|z|)
(Intercept)               0.42526    0.02106  20.197  < 2e-16 ***
sublocEXTR               -0.52036    0.03577 -14.547  < 2e-16 ***
sublocPERI               -0.44662    0.03396 -13.150  < 2e-16 ***
cellwall_typeGRAM_P       0.24321    0.01325  18.360  < 2e-16 ***
dataset_type1             0.16661    0.02140   7.787 6.84e-15 ***
sublocEXTR:dataset_type1  0.11616    0.04061   2.860  0.00423 **
sublocPERI:dataset_type1  0.20747    0.03618   5.735 9.78e-09 ***
------------------------------------------------------------------
Coefficients for variable no. 5: Comp_F
                          Estimate Std. Error z value Pr(>|z|)
(Intercept)              -0.182142   0.024236  -7.515 5.68e-14 ***
sublocEXTR               -0.126961   0.039367  -3.225 0.001259 **
sublocPERI               -0.144715   0.037589  -3.850 0.000118 ***
cellwall_typeGRAM_P       0.002639   0.015471   0.171 0.864551
dataset_type1             0.103456   0.024670   4.194 2.75e-05 ***
sublocEXTR:dataset_type1  0.040566   0.044655   0.908 0.363650
sublocPERI:dataset_type1  0.119011   0.040078   2.969 0.002983 **
------------------------------------------------------------------
Coefficients for variable no. 6: Comp_G
                         Estimate Std. Error z value Pr(>|z|)
(Intercept)               0.42861    0.02117  20.244  < 2e-16 ***
sublocEXTR                0.05704    0.03335   1.711 0.087150 .
sublocPERI               -0.04387    0.03232  -1.357 0.174716
cellwall_typeGRAM_P       0.20137    0.01314  15.321  < 2e-16 ***
dataset_type1             0.23160    0.02149  10.780  < 2e-16 ***
sublocEXTR:dataset_type1 -0.12885    0.03787  -3.403 0.000667 ***
sublocPERI:dataset_type1 -0.10985    0.03452  -3.183 0.001459 **
------------------------------------------------------------------
Coefficients for variable no. 7: Comp_H
                          Estimate Std. Error z value Pr(>|z|)
(Intercept)              -0.482376   0.026038 -18.526  < 2e-16 ***
sublocEXTR               -0.494858   0.044430 -11.138  < 2e-16 ***
```

```
sublocPERI               -0.508424   0.043229 -11.761  < 2e-16 ***
cellwall_typeGRAM_P       0.144335   0.016604   8.693  < 2e-16 ***
dataset_type1             0.016704   0.026448   0.632    0.528
sublocEXTR:dataset_type1 -0.005458   0.051221  -0.107    0.915
sublocPERI:dataset_type1  0.221745   0.046158   4.804 1.55e-06 ***
-------------------------------------------------------------------
Coefficients for variable no. 8: Comp_I
                         Estimate Std. Error z value Pr(>|z|)
(Intercept)               0.28463    0.02181  13.050  < 2e-16 ***
sublocEXTR               -0.21773    0.03586  -6.071 1.27e-09 ***
sublocPERI               -0.46035    0.03522 -13.070  < 2e-16 ***
cellwall_typeGRAM_P       0.01599    0.01395   1.146  0.25183
dataset_type1             0.17117    0.02221   7.708 1.28e-14 ***
sublocEXTR:dataset_type1 -0.07632    0.04084  -1.869  0.06163 .
sublocPERI:dataset_type1  0.11379    0.03760   3.026  0.00248 **
-------------------------------------------------------------------
Coefficients for variable no. 9: Comp_K
                          Estimate Std. Error z value Pr(>|z|)
(Intercept)              -0.018164   0.023280  -0.780   0.4352
sublocEXTR                0.004456   0.037221   0.120   0.9047
sublocPERI                0.078725   0.035012   2.249   0.0245 *
cellwall_typeGRAM_P      -0.010625   0.014475  -0.734   0.4629
dataset_type1             0.323367   0.023708  13.639   <2e-16 ***
sublocEXTR:dataset_type1 -0.436548   0.042652 -10.235   <2e-16 ***
sublocPERI:dataset_type1 -0.322125   0.037488  -8.593   <2e-16 ***
-------------------------------------------------------------------
Coefficients for variable no. 10: Comp_L
                          Estimate Std. Error z value Pr(>|z|)
(Intercept)               0.829614   0.019881  41.728  < 2e-16 ***
sublocEXTR               -0.348832   0.032798 -10.636  < 2e-16 ***
sublocPERI               -0.162749   0.030564  -5.325 1.01e-07 ***
cellwall_typeGRAM_P       0.065145   0.012852   5.069 4.01e-07 ***
dataset_type1             0.117757   0.020210   5.827 5.65e-09 ***
sublocEXTR:dataset_type1  0.007081   0.037369   0.190   0.8497
sublocPERI:dataset_type1 -0.061775   0.032724  -1.888   0.0591 .
-------------------------------------------------------------------
Coefficients for variable no. 11: Comp_M
                         Estimate Std. Error z value Pr(>|z|)
(Intercept)              -0.55664    0.02644 -21.049  < 2e-16 ***
sublocEXTR               -0.24349    0.04344  -5.605 2.08e-08 ***
sublocPERI               -0.15524    0.04142  -3.748 0.000179 ***
cellwall_typeGRAM_P       0.15531    0.01638   9.483  < 2e-16 ***
dataset_type1             0.12479    0.02686   4.645 3.39e-06 ***
sublocEXTR:dataset_type1 -0.03678    0.04958  -0.742 0.458128
sublocPERI:dataset_type1  0.08440    0.04414   1.912 0.055867 .
-------------------------------------------------------------------
Coefficients for variable no. 12: Comp_N
                         Estimate Std. Error z value Pr(>|z|)
(Intercept)              -0.20552    0.02442  -8.415  < 2e-16 ***
sublocEXTR                0.40043    0.03705  10.807  < 2e-16 ***
sublocPERI               -0.03173    0.03722  -0.853   0.3939
cellwall_typeGRAM_P      -0.06630    0.01533  -4.324 1.53e-05 ***
dataset_type1             0.14620    0.02489   5.873 4.28e-09 ***
```

```
sublocEXTR:dataset_type1 -0.08373    0.04182  -2.002   0.0453 *
sublocPERI:dataset_type1  0.02944    0.03971   0.741   0.4584
------------------------------------------------------------------
Coefficients for variable no. 13: Comp_P
                         Estimate Std. Error z value Pr(>|z|)
(Intercept)              -0.03718    0.02340  -1.589 0.112048
sublocEXTR               -0.13155    0.03761  -3.498 0.000469 ***
sublocPERI                0.14519    0.03489   4.162 3.16e-05 ***
cellwall_typeGRAM_P       0.17081    0.01470  11.618  < 2e-16 ***
dataset_type1             0.12591    0.02373   5.305 1.13e-07 ***
sublocEXTR:dataset_type1 -0.27003    0.04336  -6.227 4.75e-10 ***
sublocPERI:dataset_type1 -0.12281    0.03726  -3.296 0.000982 ***
------------------------------------------------------------------
Coefficients for variable no. 14: Comp_Q
- variable omitted (reference category) -
------------------------------------------------------------------
Coefficients for variable no. 15: Comp_R
                         Estimate Std. Error z value Pr(>|z|)
(Intercept)               0.19907    0.02222   8.958  < 2e-16 ***
sublocEXTR               -0.55539    0.03770 -14.731  < 2e-16 ***
sublocPERI               -0.28522    0.03501  -8.146 3.77e-16 ***
cellwall_typeGRAM_P       0.27854    0.01384  20.122  < 2e-16 ***
dataset_type1             0.18785    0.02250   8.348  < 2e-16 ***
sublocEXTR:dataset_type1 -0.06384    0.04315  -1.480  0.13898
sublocPERI:dataset_type1 -0.10054    0.03745  -2.685  0.00726 **
------------------------------------------------------------------
Coefficients for variable no. 16: Comp_S
                         Estimate Std. Error z value Pr(>|z|)
(Intercept)               0.19314    0.02222   8.691  < 2e-16 ***
sublocEXTR                0.28027    0.03431   8.170 3.09e-16 ***
sublocPERI                0.01254    0.03370   0.372 0.709798
cellwall_typeGRAM_P       0.05397    0.01382   3.905 9.44e-05 ***
dataset_type1             0.22255    0.02259   9.852  < 2e-16 ***
sublocEXTR:dataset_type1 -0.14335    0.03883  -3.692 0.000223 ***
sublocPERI:dataset_type1 -0.04934    0.03594  -1.373 0.169774
------------------------------------------------------------------
Coefficients for variable no. 17: Comp_T
                         Estimate Std. Error z value Pr(>|z|)
(Intercept)               0.05941    0.02260   2.628  0.00858 **
sublocEXTR                0.30739    0.03462   8.880  < 2e-16 ***
sublocPERI                0.03913    0.03440   1.137  0.25533
cellwall_typeGRAM_P       0.27592    0.01375  20.069  < 2e-16 ***
dataset_type1             0.21536    0.02296   9.382  < 2e-16 ***
sublocEXTR:dataset_type1 -0.21977    0.03932  -5.589 2.28e-08 ***
sublocPERI:dataset_type1 -0.08488    0.03671  -2.312  0.02076 *
------------------------------------------------------------------
Coefficients for variable no. 18: Comp_V
                         Estimate Std. Error z value Pr(>|z|)
(Intercept)               0.33976    0.02138  15.889  < 2e-16 ***
sublocEXTR               -0.14640    0.03445  -4.250 2.14e-05 ***
sublocPERI               -0.02227    0.03271  -0.681 0.495893
cellwall_typeGRAM_P       0.31894    0.01314  24.263  < 2e-16 ***
dataset_type1             0.25716    0.02169  11.855  < 2e-16 ***
```

```
sublocEXTR:dataset_type1 -0.14795    0.03921  -3.773 0.000161 ***
sublocPERI:dataset_type1 -0.17804    0.03496  -5.093 3.52e-07 ***
-------------------------------------------------------------------
Coefficients for variable no. 19: Comp_W
                          Estimate Std. Error z value Pr(>|z|)
(Intercept)              -1.301252   0.032594 -39.923  < 2e-16 ***
sublocEXTR                0.140722   0.049619   2.836  0.00457 **
sublocPERI                0.253109   0.047884   5.286 1.25e-07 ***
cellwall_typeGRAM_P       0.133006   0.019518   6.814 9.47e-12 ***
dataset_type1             0.044689   0.033019   1.353  0.17591
sublocEXTR:dataset_type1 -0.001911   0.056401  -0.034  0.97296
sublocPERI:dataset_type1 -0.075911   0.051069  -1.486  0.13716
-------------------------------------------------------------------
Coefficients for variable no. 20: Comp_Y
                          Estimate Std. Error z value Pr(>|z|)
(Intercept)              -0.425216   0.025491 -16.681  < 2e-16 ***
sublocEXTR                0.106453   0.039862   2.671  0.00757 **
sublocPERI               -0.050878   0.039280  -1.295  0.19523
cellwall_typeGRAM_P       0.157387   0.016087   9.783  < 2e-16 ***
dataset_type1            -0.016086   0.025970  -0.619  0.53564
sublocEXTR:dataset_type1 -0.006834   0.045497  -0.150  0.88061
sublocPERI:dataset_type1  0.206793   0.041899   4.936 7.99e-07 ***
-------------------------------------------------------------------

PRECISION MODEL:
-------------------------------------------------------------------
                          Estimate Std. Error z value Pr(>|z|)
(Intercept)               4.947631   0.013600 363.796   <2e-16 ***
sublocEXTR               -0.381077   0.019361 -19.683   <2e-16 ***
sublocPERI               -0.011332   0.021270  -0.533    0.594
cellwall_typeGRAM_P      -0.191378   0.007033 -27.212   <2e-16 ***
dataset_type1            -0.234381   0.013709 -17.097   <2e-16 ***
sublocEXTR:dataset_type1  0.432073   0.022147  19.509   <2e-16 ***
sublocPERI:dataset_type1  0.200603   0.022572   8.887   <2e-16 ***
-------------------------------------------------------------------
Significance codes: 0 ‘***’ 0.001 ‘**’ 0.01 ‘*’ 0.05 ‘.’ 0.1 ‘ ’ 1

Log-likelihood: 975721 on 140 df (945 BFGS + 3 NR Iterations)
AIC: -1951162, BIC: -1950062
Number of Observations: 19113
Links: Logit (Means) and Log (Precision)
```

Supplementary Text 2: Descriptions of partial Quasi Sequence Order (pQSO) and partial Pseudo Amino Acid Description (pPAAC)

These are two approximations of sequence order effects described by Chou in [1,2]. Suppose a protein chain of N length $R_1R_2R_3R_4R_5R_6\ldots R_N$, where R_1 represents the residue at sequence position 1, R_2 represents the residue at sequence position 2, and so forth. The sequence order effect can be approximated by a set of sequence-order-coupling numbers as defined below (1):

$$(1)\qquad \begin{aligned} \tau_1 &= \frac{1}{N-1}\sum_{i=1}^{N-1} J_{i,i+1} \\ \tau_2 &= \frac{1}{N-2}\sum_{i=1}^{N-2} J_{i,i+2} \\ \tau_3 &= \frac{1}{N-3}\sum_{i=1}^{N-3} J_{i,i+3} \\ &\ldots \\ \tau_\varphi &= \frac{1}{N-\varphi}\sum_{i=1}^{N-\varphi} J_{i,i+\varphi} \end{aligned}$$

In which τ_1 is the first-rank sequence-order-coupling number, which reflects the coupling mode between all contiguous residues along a protein sequence, τ_2 is the second-rank number that reflects the coupling mode between all the second most contiguous residues,and so on. φ is the lag value, which cannot be larger than N.

The coupling factor $J_{i,j}$ is a function of amino acids R_i and R_j, and the function chosen is the main difference between the two metrics described. For partial pQSO, the coupling factor is defined as (2):

$$(2)\qquad J_{i,j} = D^2(R_i, R_j)$$

In which $D(R_i, R_j)$ is the physicochemical distance from amino acid pairs as calculated by Schneider and Wrede[3]. For pPAAC, the coupling factor is defined as (3):

$$(3)\qquad J_{i,j} = \frac{1}{3}\{[H_1(R_j) - H_1(R_i)]^2 + [H_2(R_j) - H_2(R_i)]^2 + [M(R_j) - M(R_i)]^2\}$$

In which H_1, H_2 and M are the hydrophobicity value[4], hydrophilicity value[5] and side-chain mass values respectively, following a standard conversion in order to have a zero mean value and one standard deviation. Once all τ values have been calculated, the sequence-order effect can be described as a φ-length vector V_u (4):

$$(4)\qquad V_u = \frac{\tau_q}{\sum_{j=1}^{\varphi}\tau_j},\ (1 \le q \le \varphi)$$

In which τ_q is the qth-rank sequence-order-coupling number. Applying this formula to both types of coupling factor will result in two φ-length vectors, one for pQSO and another for pPAAC.

References

1. Chou, K. C. Prediction of protein cellular attributes using pseudo-amino acid composition. *Proteins* **43**, 246–255 (2001).
2. Chou, K. C. Prediction of protein subcellular locations by incorporating quasi-sequence-order effect. *Biochem. Biophys. Res. Commun.* **278**, 477–483 (2000).
3. Schneider, G. & Wrede, P. The rational design of amino acid sequences by artificial neural networks and simulated molecular evolution: de novo design of an idealized leader peptidase cleavage site. *Biophys. J.* **66**, 335–344 (1994).
4. Tanford, C. Contribution of Hydrophobic Interactions to the Stability of the Globular Conformation of Proteins. *J. Am. Chem. Soc.* **84**, 4240–4247 (1962).
5. Hopp, T. P. & Woods, K. R. Prediction of protein antigenic determinants from amino acid sequences. *Proc. Natl. Acad. Sci. U. S. A.* **78**, 3824–3828 (1981).
