# Supplementary material for: Ayu: a machine intelligence tool for identification of extracellular proteins in the marine secretome
Source: Nat Commun. 2025 Mar 21;16:2793. doi: 10.1038/s41467-025-57974-5 (PMC11928666; doi:10.1038/s41467-025-57974-5)
Supplement: Supplementary file 3 — Reporting Summary [file 41467_2025_57974_MOESM3_ESM.pdf]

nature portfolio

Corresponding author(s): Federico Baltar, Asier Zaragoza-Solas

Last updated by author(s): Feb 19, 2025

# Reporting Summary

Nature Portfolio wishes to improve the reproducibility of the work that we publish. This form provides structure for consistency and transparency in reporting. For further information on Nature Portfolio policies, see our Editorial Policies and the Editorial Policy Checklist.

## Statistics

For all statistical analyses, confirm that the following items are present in the figure legend, table legend, main text, or Methods section.

| n/a | Confirmed | |
|---|---|---|
| ☐ | ☒ | The exact sample size (*n*) for each experimental group/condition, given as a discrete number and unit of measurement |
| ☐ | ☒ | A statement on whether measurements were taken from distinct samples or whether the same sample was measured repeatedly |
| ☐ | ☒ | The statistical test(s) used AND whether they are one- or two-sided<br>*Only common tests should be described solely by name; describe more complex techniques in the Methods section.* |
| ☐ | ☒ | A description of all covariates tested |
| ☐ | ☒ | A description of any assumptions or corrections, such as tests of normality and adjustment for multiple comparisons |
| ☐ | ☒ | A full description of the statistical parameters including central tendency (e.g. means) or other basic estimates (e.g. regression coefficient) AND variation (e.g. standard deviation) or associated estimates of uncertainty (e.g. confidence intervals) |
| ☐ | ☒ | For null hypothesis testing, the test statistic (e.g. *F*, *t*, *r*) with confidence intervals, effect sizes, degrees of freedom and *P* value noted<br>*Give P values as exact values whenever suitable.* |
| ☒ | ☐ | For Bayesian analysis, information on the choice of priors and Markov chain Monte Carlo settings |
| ☒ | ☐ | For hierarchical and complex designs, identification of the appropriate level for tests and full reporting of outcomes |
| ☐ | ☒ | Estimates of effect sizes (e.g. Cohen's *d*, Pearson's *r*), indicating how they were calculated |

*Our web collection on statistics for biologists contains articles on many of the points above.*

## Software and code

Policy information about availability of computer code

| | |
|---|---|
| Data collection | No software was used for data collection. |
| Data analysis | Custom code used for this paper can be found at https://github.com/asierzaragoza/ayu .<br>For the statistical analysis, R 4.1.2 was used, along with the libraries ggplot2, DirichletReg, easyCODA, mgcv, statix and npmv. |

For manuscripts utilizing custom algorithms or software that are central to the research but not yet described in published literature, software must be made available to editors and reviewers. We strongly encourage code deposition in a community repository (e.g. GitHub). See the Nature Portfolio guidelines for submitting code & software for further information.

## Data

Policy information about availability of data

All manuscripts must include a data availability statement. This statement should provide the following information, where applicable:

- Accession codes, unique identifiers, or web links for publicly available datasets
- A description of any restrictions on data availability
- For clinical datasets or third party data, please ensure that the statement adheres to our policy

The code used for this paper can be found in 10.5281/zenodo.14865847 . The Tara Oceans datasets used in this study (TARA_B100001115, TARA_B100001115,TARA_B100000678, TARA_B100000678,TARA_B100001989, TARA_B100001989,TARA_B100002003, TARA_B100002003,TARA_B100000282, TARA_B100000282,TARA_Y100000031, TARA_B100000101) can be found in the European Nucleotide Archive (ENA) with project code PRJEB7988.

## Research involving human participants, their data, or biological material

Policy information about studies with human participants or human data. See also policy information about sex, gender (identity/presentation), and sexual orientation and race, ethnicity and racism.

| | |
|---|---|
| Reporting on sex and gender | The data used in this study does not include human data nor human participants. |
| Reporting on race, ethnicity, or other socially relevant groupings | The data used in this study does not include human data nor human participants. |
| Population characteristics | The data used in this study does not include human data nor human participants. |
| Recruitment | The data used in this study does not include human data nor human participants. |
| Ethics oversight | No Ethics oversight was required for this study. |

Note that full information on the approval of the study protocol must also be provided in the manuscript.

# Field-specific reporting

Please select the one below that is the best fit for your research. If you are not sure, read the appropriate sections before making your selection.

☐ Life sciences ☐ Behavioural & social sciences ☒ Ecological, evolutionary & environmental sciences

For a reference copy of the document with all sections, see nature.com/documents/nr-reporting-summary-flat.pdf

# Ecological, evolutionary & environmental sciences study design

All studies must disclose on these points even when the disclosure is negative.

| | |
|---|---|
| Study description | The study was based on the analysis of publicly available protein databases (UniProt) and marine metagenomes/metatranscriptomes (Tara Oceans). |
| Research sample | *Describe the research sample (e.g. a group of tagged Passer domesticus, all Stenocereus thurberi within Organ Pipe Cactus National Monument), and provide a rationale for the sample choice. When relevant, describe the organism taxa, source, sex, age range and any manipulations. State what population the sample is meant to represent when applicable. For studies involving existing datasets, describe the data and its source.* |
| Sampling strategy | We used three samples from each depth from the Tara Oceans dataset, all belonging to the same oceanic region. We considered that three samples were enough, as we were analyzing these samples as a means of showing the capabilities of our tool. |
| Data collection | Data were downloaded from the ENA website. |
| Timing and spatial scale | Not applicable. |
| Data exclusions | All data from the metagenomes / metatranscriptomes were used. From the uniprot dataset, only the proteins that did not meet the annotation requirements were excluded, as stated in the Methods section. |
| Reproducibility | All analyses on this paper are computer-based and enough information has been provided in materials and methods to reproduce the results. |
| Randomization | Only relevant to the training/testing splits on the machine learning algorithm, which were performed using GraphPart. |
| Blinding | Only relevant to the training/testing splits on the machine learning algorithm, which were performed using GraphPart without further modification. |

Did the study involve field work? ☐ Yes ☒ No

# Reporting for specific materials, systems and methods

We require information from authors about some types of materials, experimental systems and methods used in many studies. Here, indicate whether each material, system or method listed is relevant to your study. If you are not sure if a list item applies to your research, read the appropriate section before selecting a response.

## Materials & experimental systems

| n/a | Involved in the study |
|---|---|
| ☒ | ☐ Antibodies |
| ☒ | ☐ Eukaryotic cell lines |
| ☒ | ☐ Palaeontology and archaeology |
| ☒ | ☐ Animals and other organisms |
| ☒ | ☐ Clinical data |
| ☒ | ☐ Dual use research of concern |
| ☒ | ☐ Plants |

## Methods

| n/a | Involved in the study |
|---|---|
| ☒ | ☐ ChIP-seq |
| ☒ | ☐ Flow cytometry |
| ☒ | ☐ MRI-based neuroimaging |

# Plants

| | |
|---|---|
| Seed stocks | *Report on the source of all seed stocks or other plant material used. If applicable, state the seed stock centre and catalogue number. If plant specimens were collected from the field, describe the collection location, date and sampling procedures.* |
| Novel plant genotypes | *Describe the methods by which all novel plant genotypes were produced. This includes those generated by transgenic approaches, gene editing, chemical/radiation-based mutagenesis and hybridization. For transgenic lines, describe the transformation method, the number of independent lines analyzed and the generation upon which experiments were performed. For gene-edited lines, describe the editor used, the endogenous sequence targeted for editing, the targeting guide RNA sequence (if applicable) and how the editor was applied.* |
| Authentication | *Describe any authentication procedures for each seed stock used or novel genotype generated. Describe any experiments used to assess the effect of a mutation and, where applicable, how potential secondary effects (e.g. second site T-DNA insertions, mosiacism, off-target gene editing) were examined.* |
