# Supplementary material for: Ayu: a machine intelligence tool for identification of extracellular proteins in the marine secretome
Source: Nat Commun. 2025 Mar 21;16:2793. doi: 10.1038/s41467-025-57974-5 (PMC11928666; doi:10.1038/s41467-025-57974-5)
Supplement: Supplementary file 4 — Description of Additional Supplementary Files [file 41467_2025_57974_MOESM4_ESM.pdf]

## Description of Additional Supplementary Files

Supplementary Data 1

Tara Oceans datasets used in this study.

Supplementary Data 2:

Marine organisms selected for the training and validation dataset.

Supplementary Data 3:

Protein features used in Ayu.
